# Supplementary material for: Performance of early warning signals for disease re-emergence: A case study on COVID-19 data
Source: PLoS Comput Biol. 2022 Mar 30;18(3):e1009958. doi: 10.1371/journal.pcbi.1009958 (PMC9000113; doi:10.1371/journal.pcbi.1009958)
Supplement: S1 Text — Sec A: Mathematical models and assumptions. Sec B: Data collection and curation. Sec C: Estimating R(t) with Bayesian inference using MCMC. Sec D in S1 Text: Determining the rate of approach for R(t) → 1. Sec E: Evolution of EWS for all countries. Sec F: ARIMA detrending and corresponding global EWS. Sec G: Further investigation on AUC values. Sec H in S1 Text: Analysis of incidence data. Fig A: theoretical EWS for epidemic re-emergence. Fig B: Examples of discarded time series. Fig C: Curves of active cases for the considered countries. Fig D: Estimate of transition rate to R critical value. Fig E: Evolution of the considered indicators for all countries. Fig F: Investigation of variance for countries in N. Fig G: Analysis of the dynamical characteristics of the countries included in the data set, for incidence data. Fig H: Analysis of the residuals from the detrending methods. Fig I: Analysis of the variance in the Luxembourg setting. Fig J: ROC curves for each considered indicator. Fig K: Evolution of EWS far from the transition point. Table A: Additional information about the selected countries. Table B: ARIMA model parameter combinations. Table C: AUC scores for different indicators. Table D: ARIMA model parameter combinations over incidence data. (PDF) [file pcbi.1009958.s001.pdf]

Supplementary Material for:
Performance of early warning signals for disease re-emergence: a case study on COVID-19 data

Daniele Proverbio[1,2*], Françoise Kemp[1], Stefano Magni[1], Jorge Gonçalves[1,3]

1 Luxembourg Centre for Systems Biomedicine, University of Luxembourg, Belvaux, Luxembourg
2 College of Engineering, Mathematics and Physical Sciences, University of Exeter, Exeter, United Kingdom
3 Department of Plant Sciences, University of Cambridge, Cambridge, United Kingdom

* daniele.proverbio@uni.lu

A Mathematical models and assumptions

As described in the Main Text, early warning signals (EWS) are measures that rest upon modelling assumptions. To clarify the most relevant assumptions, which are tested in the present study, we recall how EWS are theoretically derived.

When consistent with a mean-field approximation, the dynamics of COVID-19 infectiousness is well described by SIR-like models [1, 2]. To illustrate how early warning signals can be subsequently derived, we here recall the process described in [3]. SIR models describe disease processes in homogeneous populations of susceptible individuals (X), which can get infectious (Y) and eventually removed (Z) by death or recovery. In the deterministic SIR, transitions among states are governed by the infection rate β and the removal rate γ, which lumps recovery and death rate. Empirical values for COVID-19 can be traced in the abundant literature, e.g. [4]. In addition, systems are often open with influx rate μ' and outflux rate μ'' of people. However, many travelling restrictions were in place during the year 2020 (see ACAPS website (`https://bit.ly/3nFFqUS`) for a dataset of government measures.). Hence, we can assume that such fluxes are small and balanced: $\mu' = \mu'' = \mu$. Along with that, we model an influx rate of new cases η that can trigger subsequent disease outbreaks. Finally, intervention measures introduce a probability p that some susceptible individuals are isolated and protected, either physically (e.g. through non-pharmaceutical interventions limiting social interaction or through changes in people's behaviour) or by vaccination [5]. The model reads:

$$\begin{aligned}
\dot{X} &= \mu(1-p) - \beta XY - (\eta+\mu)X \\
\dot{Y} &= \beta XY + \eta X - (\gamma+\mu)Y \\
\dot{Z} &= \mu p + \gamma Y - \mu Z\,.
\end{aligned} \tag{A}$$

It is assumed that the population size N is constant, that is $X + Y + Z = 1$ since variables are normalized. In this case, the control parameter R is given by [3]:

$$R = \frac{\beta}{\gamma+\mu}(1-p) \tag{B}$$

and reaches its critical value 1 for $p^* = 1 - (\gamma + \mu)/\beta$, at which the dynamics undergo a transcritical bifurcation on the (Y, p) bifurcation diagram [3]. We remark that we consider here an example from literature: R might change in time after being driven by other evolving parameters such as one that tunes the contact rate β [2]. Without protection and extra fluxes, the basic reproduction number for COVID-19 was estimated at the beginning of the pandemic in the range $2 < R < 4$ (*cf., e.g.,* [6]).

If $p(t)$ changes slowly over time, we can mathematically express the SIR model A approaching the transition as a slow-fast system:

$$\begin{cases} \text{Eq. } A \\ \dot{p} = \epsilon f(X, Y, p) \end{cases} \tag{C}$$

where $0 < \epsilon \ll 1$ and f is a function that describes its change. A limit case is $\epsilon \to 0$. This condition is necessary to interpret the dynamical shift as a slow crossing through a bifurcation point, and to compute its associated summary statistics [7]. Often [3,8], the function f is assumed to be a constant $f = \tilde{p}$. This way, the protection probability is, at first approximation, a linear function of time:

$$p = p_0 + \tilde{p}t\,, \tag{D}$$

and R as well, following Eq. B. If $\tilde{p} > 0$, then R gets reduced and, if it was above 1, the bifurcation is crossed from above towards elimination. If $\tilde{p} < 0$, R increases and, if it was below 1, the bifurcation is crossed from below, towards a new emergence. Only the second case can be investigated with COVID-19 data, as most countries implemented suppression measures very rapidly [9] and thus Eq. C is usually not satisfied in the first case.

When the transition is approached from below, and if there are few cases, stochastic fluctuations are not negligible. Hence, we need to consider the transitions described by a stochastic master equation. Reducing the stochastic master equation to Eq. C and a Fokker-Plank equation for the fluctuations was already performed in [3,10]. Hence, we briefly recall them to illustrate the assumptions underlying the behavior of early warning signals prior to the transition.

First, note that system A, along with condition $\dot{N} = 0$, can be reduced to its first two equations. Hence, we just need to consider the transitions in and out X and Y. Second, for each small time step dt, the quasi-steady state p is constant.

Transitions in and out states are described as random jump processes. Such states are (X, Y), $(X - 1, Y)$, $(X, Y + 1)$ and so on. Using $\alpha = (X, Y)$ to describe the "basic" state, $\bar{\alpha}$ is any other state and $P(X, Y, t) = \text{Prob}(X(t), Y(t) = (x, y))$ is the probability that the state vector is equal to some pair of non negative integer numbers (x, y). Finally, $T_i(\alpha, \bar{\alpha})$ is the transition probability between states, and is a function of transition rates. The subscript i denotes all possible jumps in and out of the states. Examples of $T_i(\alpha, \bar{\alpha})$ are found in [3,11], depending on the system of interest. Consequently, the master equation for the stochastic process is:

$$\frac{dP(\alpha, t)}{dt} = \sum_{\alpha \neq \bar{\alpha}} T_i(\alpha|\bar{\alpha})P(\bar{\alpha}, t) - \sum_{\alpha \neq \bar{\alpha}} T_i(\bar{\alpha}|\alpha)P(\alpha, t) \tag{E}$$

In general, Eq. E is nonlinear. To have analytical results about its average behavior and the fluctuations around it, the van Kampen expansion can be used [12] to approximate the discrete random variables with continuous random variables. This depends on having large N, which holds in our case when we consider the population of medium to big countries. To leading order, the expansion of Eq. E is equivalent to Eq. C. To quantify the fluctuations at next-to-leading order, the obtained Fokker-Plank

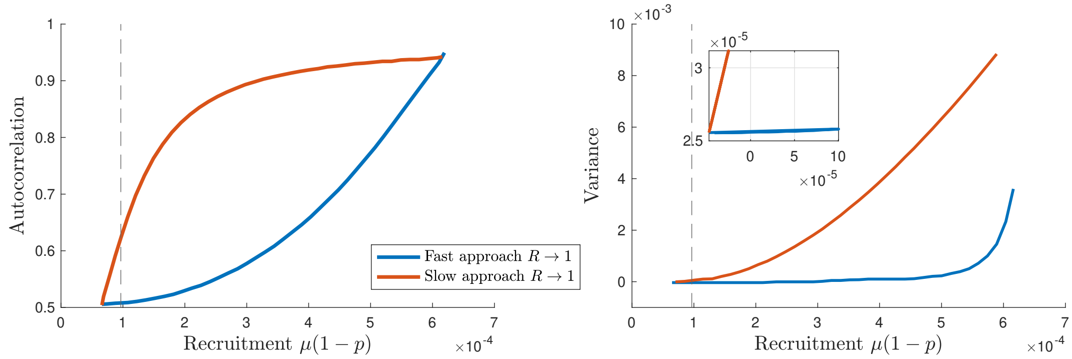

Fig A. Theoretical EWS for epidemic re-emergence. Left: the lag-1 autocorrelation increases before the transition if the approach of R to 1 is slow (slow-fast assumption satisfied, red); otherwise it increases after the transition (bifurcation delay, blue). Right: the variance increases before the transition (dashed grey line) if the approach of R to 1 is slow (slow-fast assumption satisfied, red; the inset magnifies the effect). Otherwise, it increases after the transition (bifurcation delay, blue). The plots are derived from the analytical results of [3] and reproduce its Fig 9, d—e. On the x-axis: values for the recruitment rate $[d^{-1}]$ of new infectious that could trigger a re-emergence.

equation is equivalent to the following system of stochastic differential equations [10]:

$$\begin{aligned} \frac{d\sigma}{dt} &= b_{11}\sigma(t) + b_{12}\zeta(t) + \Gamma_1(t) \\ \frac{d\zeta}{dt} &= b_{21}\sigma(t) + b_{22}\zeta(t) + \Gamma_2(t) \end{aligned} \tag{F}$$

where Γ_j are white noise processes and the elements of the matrix $B = \{b_{kl}\}$ are functions of transition rates. Eq. F connects the stochastic description of the epidemic with SIR-like models like Eq. A would with a noise term.

Eq. F can be analysed by its Fourier transform. By considering the fluctuations around the infectious state, we can derive the power spectrum and, through integration, the variance, the autocorrelation and other statistical moments. Their specific values depend on the eigenvalues of matrix B and of the covariance matrices of Γ_j. The evolution of variance and autocorrelation next to the transition, as obtained in [3], is shown in Fig A for a slow and a fast approach to $R(t) = 1$. The trend of these summary statistical indicators on the fluctuations, prior to the transition, constitutes the set of signals that could detect the transition itself; for instance, the increase in variance, often measured in terms of Kendall's τ. The Kendall's τ score is a non-parametric measure of ranks correlation, which is usually used to identify increasing trends [13, 14]. Such increasing trends are known in the literature as early warning signals (EWS) [15].

Finally, let us recall the general theory of EWS on bifurcations. Any system approaching a transcritical bifurcation is, in its vicinity, topologically equivalent to a transcritical normal form [16, 17]. This is a minimal model that retains the systems' behavior and resilience properties in the vicinity of a bifurcation. Models can be transformed to a normal form after an appropriate change of variables [18]. The normal form associated to a transcritical bifurcation has the form:

$$\dot{\theta} = q\theta - \theta^2 \tag{G}$$

where q is the bifurcation parameter and θ the variable of interest (Y from eq. A, in this specific case). This form represents a system whose extinction state and positive steady state coalesce and exchange stability when q reaches its critical value. If there are statistical fluctuations ξ, we can write their evolution as a linearization around the

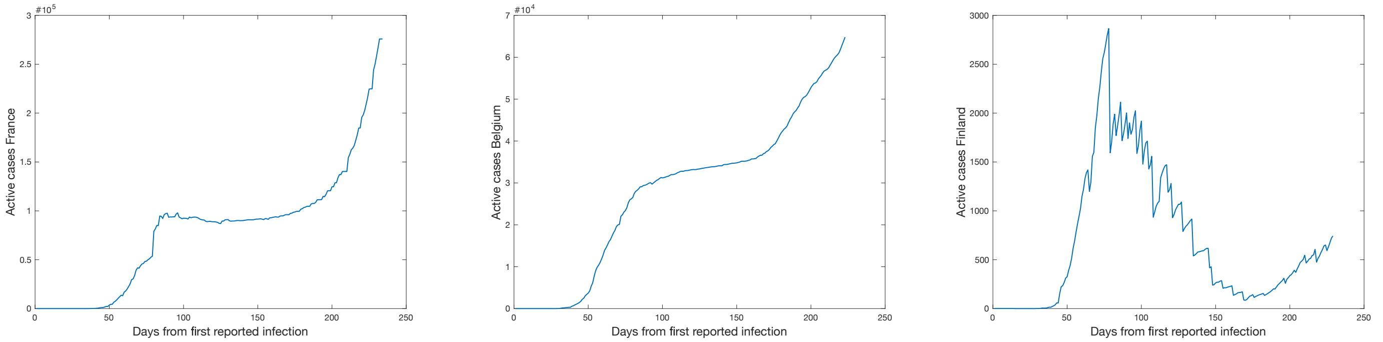

Fig B. Examples of discarded time series, following some of the criteria explained in "Methods and Mathematical Theory" of Main Text. Among others, France and Belgium had active cases curves that differ from the typical bell-shaped SEIR-like behavior. This is related to reporting of recovered and dead patients. On the other hand, Finland is an example of sawtooth evolution, due to recovered cases being reported with different frequencies than daily cases. Data from [25].

equilibrium $\tilde{\theta}$, thus resulting in a Langevin equation [7,19]:

$$d\xi = -\frac{\partial f}{\partial \theta}\Big|_{\tilde{\theta}}\xi + \sqrt{\sigma^2 g^2(\tilde{\theta})}dW \tag{H}$$

where dW is a Wiener process, σ^2 models the noise level and g is the diffusion coefficient of the associated Fokker-Planck equation. With a linear $|\partial f/\partial\theta|_{\tilde{\theta}}| = k$, Eq. H is an Ornstein–Uhlenbeck process with known statistical moments [20]. Hence, we can complement the theoretical results described above with those from the theory of statistical indicators of normal forms, e.g. [7,19,21]. Important remarks are that: a) multiplicative noise can modify the indicators trend, e.g. by making it decrease; b) EWS are expected to work best in the vicinity of the transition; c) there can be bifurcation delays associated to out-of-equilibrium phenomena, i.e. changes of the system state (and of its indicators) that lag behind the bifurcation of the limit case.

B Data collection and curation

Among all the countries that registered a re-emergence of COVID-19 epidemic between April and September 2020, we first selected those for which meaningful prevalence data could be directly obtained from official sources or reconstructed with Eq. 4 from Main Text. Examples of discarded data series are reported in Fig B. We recall the underlying hypothesis of this study: that dynamical early warning signals are expected to work when the investigated system can be described by a proper dynamical model. Hence, we did not consider time series for which active cases do not display the typical SEIR-like behavior like that described in [2,22–24] (mostly due to data management and reporting), or for which recovered cases are reported with different frequencies, resulting in sawtooth curves for active cases. In the later case, the detrended fluctuations would be associated to reporting standards and would not be representative of dynamical fluctuations associated to EWS. Other selecting criteria to increase the quality of the dataset are discussed in "Methods and Mathematical Theory" of the Main Text.

Information about the specific restrictions in place was not directly used as we concentrated on their lumped effect captured by $R(t)$. Nonetheless, it is possible to verify on the ACAPS database (`https://www.acaps.org/covid19-government-measures-dataset`) that all considered countries issued a lockdown of varying intensity and additional measures, to push $R(t)$ below 1 after the first wave. ACAPS is an independent, non-profit

information provider helping humanitarian actors to respond more effectively to disasters. The ACAPS analysis team has aggregated and classified interventions from different sources (media, governments and international organizations), for all countries and in time.

Curves of active cases for all countries considered in Main Text, their smoothing and the associated $R(t)$ are displayed in Fig C. The smoothed curves serve as basis for the detrending, the analysis of the noise distribution of each country and the subsequent investigation of early warning signals, as explained in Main Text. Specific information about population, surface and testing strategy of the considered countries is also reported in Tab. A.

Country	Population	Area $[km^2]$	Avg. daily tests/1000 inh.	Share positive tests
State of Victoria (AUT)	6.681	227.444	1.8	<0.1%
Austria	8.917	83,879	0.6	[0.1; 0.6]%
Denmark	5.831	42,933	2.0	[0.1; 1]%
Israel	9.217	22,145	1.9	[0.3; 2]%
Japan	125.8	377,975	0.1	[1; 3.9]%
Korea, South	51.78	100,210	0.15	[0.3; 0.7]%
Luxembourg	0.632	2,586	10	[0.1; 0.4]%
Nepal	29.14	147,516	0.2	[0.3; 4.7]%
Singapore	5.686	728.6	5.1	[1.7; 3.7]%
Veneto (ITA)	4.906	18,345	0.7	[0.4; 2.7]%

Table A. Additional information about the selected countries: population (in millions inhabitants), area (in km^2), average number of daily tests per 1000 inhabitants, performed during the considered period (March - August 2020) and share of positive tests (in the same considered period, in percentage range from min to max values). The last two indicators derive from [26], to which we refer for the full time evolution. As mentioned in the Main Text, Luxembourg stands out for its higher number of tests performed per inhabitant.

C Estimating R(t) with Bayesian inference using MCMC

Following standard methodologies [27, 28], we reconstruct the day-by-day evolution of the reproduction number $R(t)$ by fitting a Poisson transmission model with Markov Chain Monte Carlo (MCMC) methods.

When modelling "arrivals" of discrete-state stochastic processes (*cf.* section S1), Poisson processes are widely employed. For instance, they effectively modeled the transmission of Ebola [29] and Influenza [30]. Given an average rate of λ new cases per day, the probability of seeing k new cases is distributed according to the Poisson distribution:

$$p(k|\lambda) = \frac{\lambda^k e^{-\lambda}}{k!} \,. \tag{I}$$

In turn, λ depends on R as [31]:

$$\lambda = k_{t-1} e^{\gamma(R-1)} \tag{J}$$

for all time points. γ is the reciprocal of the serial interval, which is around 4 days for COVID-19 [32, 33]. To account for such uncertainty, we treat γ as a random sample from a Gaussian distribution centered in 4 days with an assumed standard deviation of 0.2. Hence, the probability of observing a time series $x = \{x_t\}$ for each t between t_0 and T discretised by a small step δ is given by:

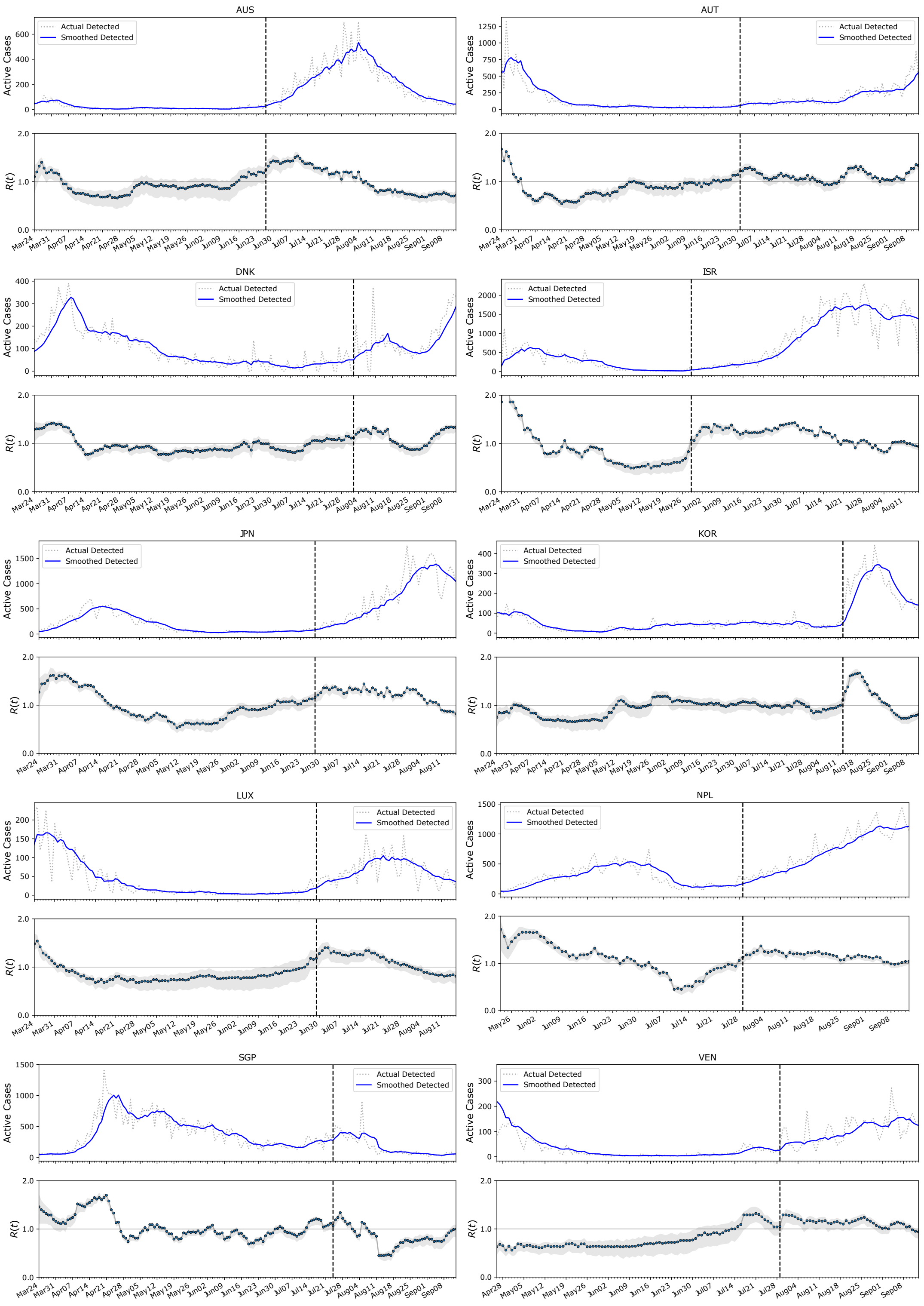

Fig C. Curves of active cases for the considered countries, along with their associated $R(t)$ (median values and 50% credible intervals). The vertical dashed line identifies the day marked for the transition an reported in Table 1 of Main Text.

$$p(x|R) = \prod_{t=t_0}^{T-\delta} p(k_{t+\delta}|\lambda_t) \tag{K}$$

Before t_0, no case was reported. Following Bayes' rule, the posterior distribution of R, for each time point, is given by (up to a normalization constant):

$$p(R|x) \propto p(x|R)q(R), \tag{L}$$

where $q(R)$ is a prior distribution. For each time point after $t_0 + 1$, the prior equals the preceding posterior. We follow the implementation of the Rt-live web service (`https://github.com/rtcovidlive/covid-model`) to generate thousands of MCMC samples with the Metropolis Hastings algorithm, starting with a Gaussian prior $\mathcal{N}(R, \sigma)$. We assumed $\sigma = 0.15$. From the posterior distribution, we also estimated the probability that $R(t)$ is greater than 1, which was in turn used to define the "ground truth" date of regime transition for the epidemic trend (see also "Methods and Mathematical Theory" of Main Text for further discussion). Fig C shows the results of the Bayesian $R(t)$ estimation (median values and 50% credible intervals) for the considered countries.

D Determining the rate of approach for $\mathbf{R(t) \to 1}$

As explained in the Main Text, we are interested in evaluating how rapidly the transition point is approached, for each country. To do so, the simplest linear trend

$$y = a + b \cdot x, \tag{M}$$

corresponding to Eq. 6 in Main Text, is assumed and fitted to $R(t)$ time series, obtained as described above and reported in Fig C. The estimated regression coefficient is informative about the rate of approach of $R(t) \to 1$.

The fitting was performed with *scipy.optimize* routine, considering as uncertainty the 50% credible interval from the distribution of $R(t)$. The goodness of fit was evaluated with the reduced χ^2 score. Results are displayed in Fig D. The $\chi^2_{\text{red}} < 1$ guarantees the goodness of the linear fit, which allows to extrapolate the regression coefficient as a measure of the rate of $R(t) \to 1$. The values of $b \pm \sigma_b$ are then reported in Fig 1C of Main Text.

E Evolution of indicators for all countries

In this section, we show and discuss the evolution of the considered early warning indicators for all countries, including those that are not shown in Fig 4 of Main Text.

Let us first consider EWS obtained from Gaussian filter detrending. In Fig E we observe the evolution of the indicators, either globally (Ea) or locally, just prior to the bifurcation (Eb for the variance, the most robust one as discussed in Main Text). We can observe the patterns discussed in the Main Text, associated to the different countries belonging to the test set $\mathcal{Y}$ or not ($\mathcal{N}$). Within $\mathcal{Y}$, the variance increases prior to the transition and gives very few spurious signals before, whereas other indicators can be more misleading when the transition still did not happen. Overall, predicted trends of EWS prior to the bifurcation are associated to satisfied theoretical assumptions such as gradual approach of $R(t) \to 1$ and white noise (*cf.* Fig 1 in Main Text and discussion thereafter). Not satisfying these requirements might disrupt the expected increasing trend and results in misleading signals, see in Fig E the countries listed in $\mathcal{N}$. Hence, if a system is not known or there is difficulty in determining the type of data, incorrect conclusions could be drawn when interpreting the time series trend. Fig 5 and Table 2 of Main Text quantify the performance of EWS for all countries.

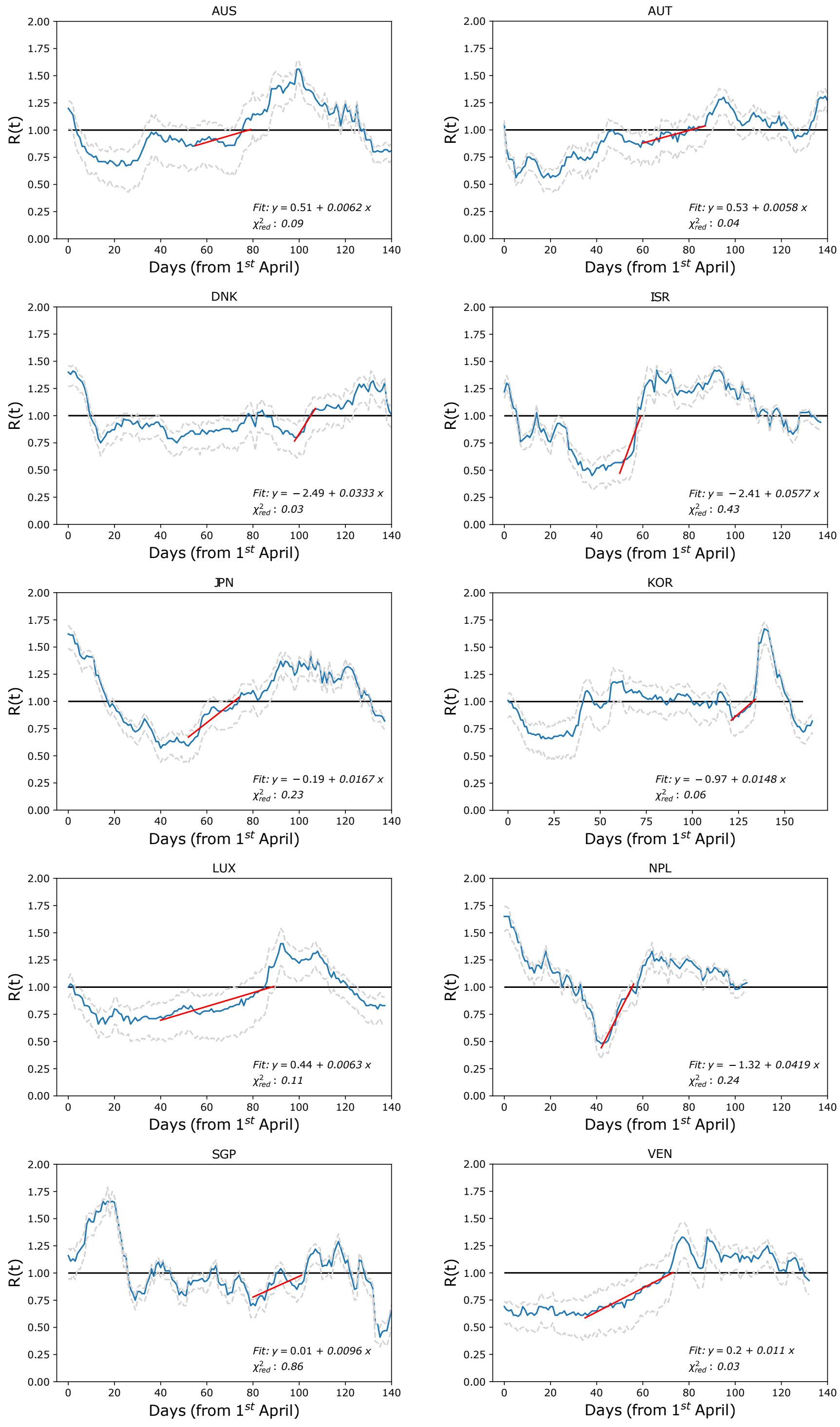

Fig D. Estimate of transition rate to R critical value. Fitting $R(t)$ evolution (blue line; dashed lines are ±50% CI) with a linear trend prior to the transition estimates the rate of approach to the threshold value 1. The fit begins around the minimum of $R(t)$ (excluding small fluctuations) until when the median value crosses 1 (horizontal line). The best fit curve is in red. $\chi^2_{\text{red}} < 1$ guarantees that a good fit is achieved for the simple linear function Eq. M. The regression coefficient b is a proxy for the rate of approach to 1. Its associated uncertainty is not reported here but is shown in Fig 1 of Main Text.

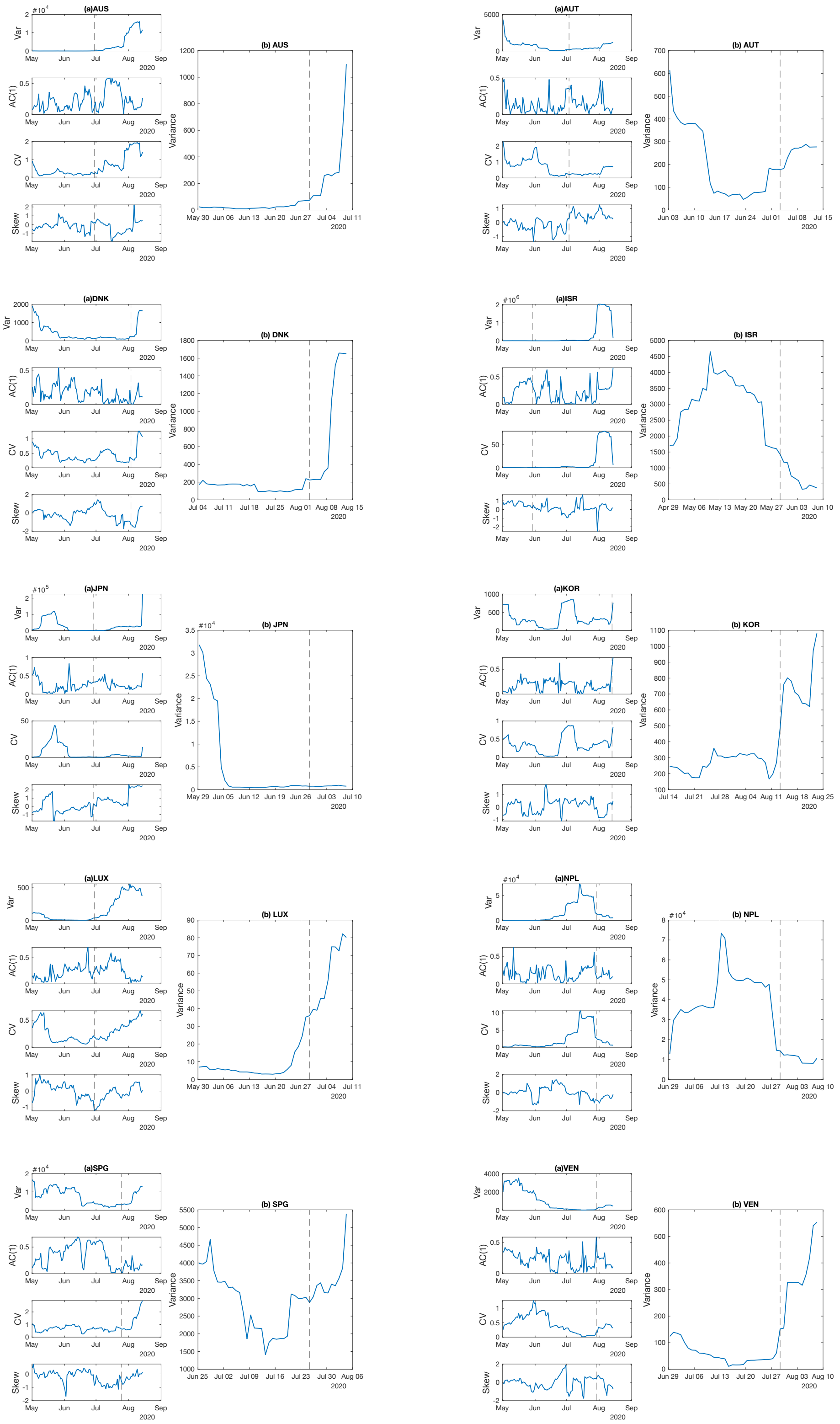

Fig E. Evolution of the considered indicators for all countries. Figs (a) report their global evolution, from the end of the first wave to the second. Figs (b) focus on the behavior of the variance close to the transition (local behavior). As discussed in the Main Text, we report the values of indicators after a moving window of size 14 days, associated to the rightmost data point to avoid "looking into the future". The vertical dashed line marks the transition point.

F ARIMA detrending and corresponding global EWS

As explained in the Main Text, on top of moving average smoothing and Gaussian kernel filtering, we tested the ARIMA detrending method. ARIMA (Auto Regressive Integrated Moving Average) is an automatic method to identify the leading trends of a time series [34], depending on three terms (p, q, d) that need to be adjusted for each data set. Initially, the d term is identified by checking for stationarity in the data. Then, the other terms are automatically identified with the python function *pmdarima* for ARIMA estimation. Table B summarises the selected ARIMA as well as their residuals mean and standard deviation, for each country.

Country	ARIMA parameters	Residuals mean	Residuals std
State of Victoria (Australia)	(0,2,1)	-2.51	197.9
Austria	(1,2,0)	2.58	121.5
Denmark	(0,2,1)	3.17	86.97
Israel	(3,2,2)	34.21	823.5
Japan	(1,2,2)	-0.279	349.12
Korea, South	(3,1,3)	6.462	141.14
Luxembourg	(2,1,3)	0.340	30.90
Nepal	(1,2,0)	0.248	241.66
Singapore	(1,2,0)	-0.070	152.47
Veneto (Italy)	(0,2,1)	0.133	70.048

Table B. ARIMA model parameter combinations over prevalence data, residuals mean and standard deviation, for each country.

G Further investigation on AUC values

Table 2 of Main Text reports AUC values close to 0 for variance and CV for the $\mathcal{N}$ test set, in particular after ARIMA detrending. This would correspond to good detection performance for decreasing trends of such indicators, which would contradict the theoretical results. We have carried out a further investigation to address this oddity. In Fig F we an observe that a decreasing variance is indeed observed close to the transition of $\mathcal{N}$ countries. Zooming out, we nonetheless observe that this corresponds to one of the following situations: a continuation of a decreasing trend after the first wave or fluctuations is a rather stable trend, eventually followed by a delayed increase after the transition. We hypothesise that these features are again associated with the rapid re-emergence and the noise distribution. On the one hand, the indicator had little time to settle to stable trends and then increase again; on the other hand, there could be some bifurcation delay playing a role. Hence, we suggest that this is a spurious effect. These observations calls for caution when interpreting the AUC values related to decreasing trends and reminds of the importance to compare the results with theoretical predictions.

H Analysis on incidence data

As mentioned in the Main Text, we complement the analysis on prevalence data with that on incidence data (daily new cases). The latter avoids potential bias induced by the estimation of recovered patients, but might be more sensitive to systematic

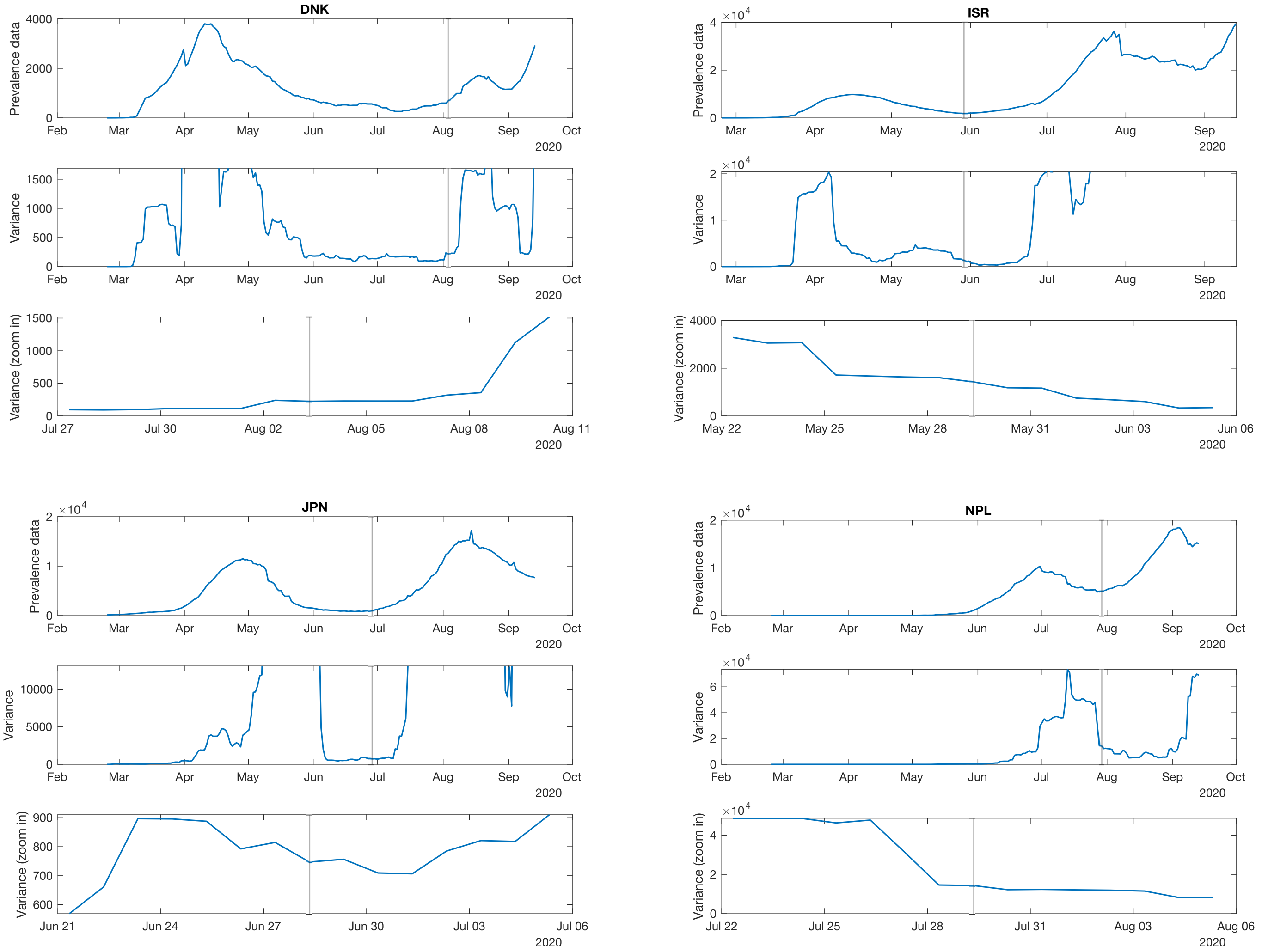

Fig F. Investigation of variance for countries in $\mathcal{N}$. For each country, the top panel reports the prevalence data, the middle one is for the full time series of variance, while the bottom one zooms in the vicinity of the transition. Note that ISR and JPN were zoomed to better see the variance trends; when it's not visible anymore, the variance reached higher values that those encompassed in the y-axis scale.

fluctuations associated with testing routines. Hence, the two analysis can be regarded as representative of real-world monitoring capacities.

The analysis is performed in the same way as the one described in Main Text; the indicators and methodologies are also consistent. Therefore, we refer to the Main Text (Methods and figures corresponding to the ones presented here) for methodological explanations.

In accordance with preliminary studies that investigated incidence data for re-emergence of infectious diseases [11, 35], the results here obtained are similar to what observed for prevalence data. However, there is a number of differences worth stressing. In the remaining of this section, we will highlight both similarities and differences.

To begin with, the distribution of fluctuations around the average trend is different than what observed in Fig 1 of the Main Text. In the case of incidence data, the deviation from Gaussian noise distribution is more pronounced (see Fig Gb), which

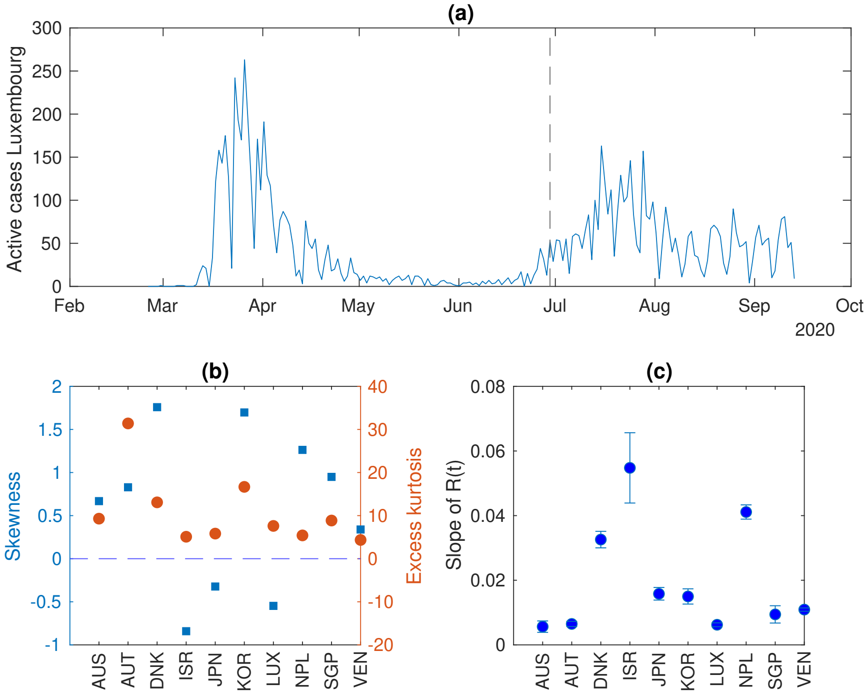

Fig G. Analysis of the dynamical characteristics of the countries included in the data set, for incidence data. a) An example of an epidemiological curve of daily new cases from Luxembourg. The dashed line indicates the transition, measured by $R > 1$. b) Measures of the distribution of data fluctuations. Skewness μ indicates the symmetry of the distribution, whereas excess of kurtosis $\gamma - 3$ indicates the relevance of its peak with respect to the tails. Large deviations from $\mu = 0$ (dashed line) and $\gamma = 3$ are associated with non-normal distributions. c) The regression coefficient of $R(t)$ and its associated uncertainty.

reflects the larger fluctuations usually associated with testing protocols like reduced weekend testing (an example is shown in Fig GA). Interpreting the subsequent results as consequences linked to the noise distribution is therefore more challenging. In addition, we observe (Fig GB) that Japan and South Korea have different skewness and excess of kurtosis than what reported in Fig1 of Main Text. Therefore, as the rate of approach to $R(t) = 1$ is conserved (Fig GC), we follow the initial criterion of placing one country in the test set $\mathcal{Y}$ and one in $\mathcal{N}$, but we swap them: Japan is placed in $\mathcal{Y}$ and South Korea in $\mathcal{N}$. We do this for consistency with the previous analysis, but we also verified that the following results are little sensitive to this choice.

Next, we verify that the detrending methods provide consistent results to those observed in the Main Text. Moving average and Gaussian filtering are similar to one another (Fig H); in this case, the ARIMA is notably better correlated with the other two methods, possibly due to the short-term fluctuations being mostly driven by systematic testing routines (compare the smoother trend in Fig 1A with the weekly oscillating pattern in Fig GA) that are smoothed by all filtering procedures. In addition, the local behavior of the variance shows an increasing trend, as expected from preliminary theoretical studies (Fig I); differently from the case with prevalence data, both the eyeball visualization and the Kendall's τ are very similar between ARIMA and Gaussian filtering results. We recall that we first perform this study on Luxembourg, as the Large Scale Testing scheme [36] in place suggests that it is the mostly controlled setting.

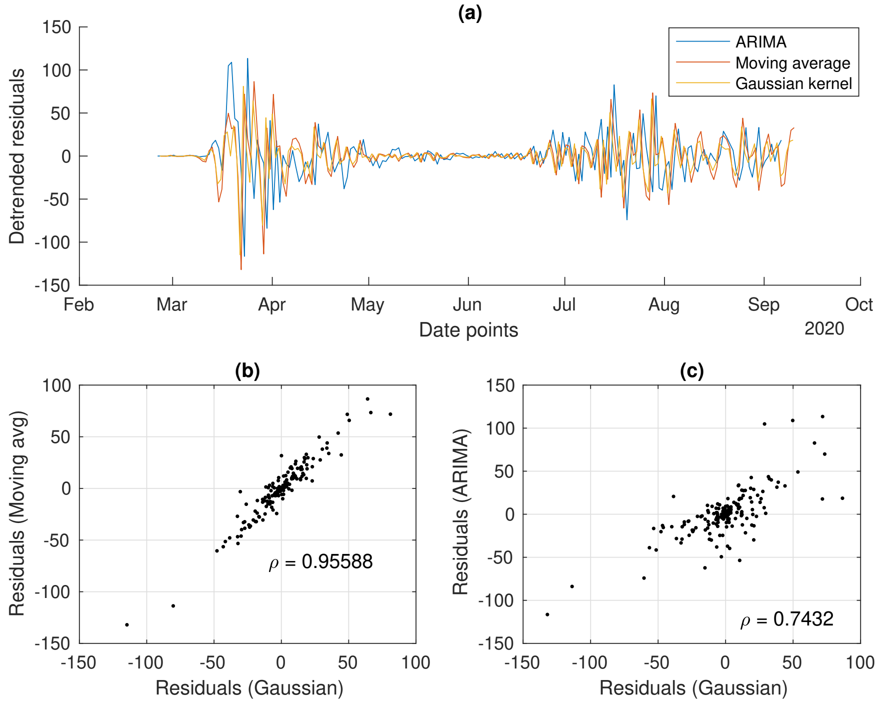

Fig H. Analysis of the residuals from the detrending methods (one case study from Luxembourg shown). a) The detrended fluctuations time series. b) Correlation between residuals obtained from Gaussian or moving average filtering. c) Correlation between residuals obtained from Gaussian or ARIMA filtering.

The qualitative global behavior of statistical indicators for EWS is also similar to what observed in the case of prevalence data (Fig K). For the same countries used in Fig 4 of Main Text, we observe an increase of the variance prior to the transition and potentially spurious fluctuations of the AC(1) and CV, likely associated to moving window effects and fluctuations of the raw data. In contrast, the skewness seems to follow a more pronounced increasing trend. Likewise for the Main Text, these eyeball observations are subsequently quantified with ROC and AUC analysis.

From the quantitative analysis about sensitivity and specificity of the indicators in detecting the transition, we observe overall similar results to those reported in the Main Text: the AC(1) and CV are worse than a random classifier even for countries in $\mathcal{Y}$, whereas the variance provides more robust results (Fig J and Table C). However, we observe a number of interesting differences over both ARIMA and Gaussian filtering results. To begin with, variance and CV on the $\mathcal{N}$ set are less associated with decreasing trends close to the transition but are closer to the performance of random classifiers. This might suggest that, as discussed in the Main Text, the result on prevalence data was likely spurious and related to the decreasing of the first wave. As daily new cases do not have a time delay due to recovered cases, such effect is indeed less marked. The second, more striking difference regards the skewness. As shown in Fig J, and quantified in Table C, the skewness is particularly good in detecting the transition to disease emergence on the test set $\mathcal{Y}$. This observation differs from what anticipated in [11] but is more in line to what was suggested in [37]. Since there are only few studies connecting observed EWS to noise distribution (like the one observed in Fig G), we cannot make a conclusive interpretation of this fact. Nonetheless, we

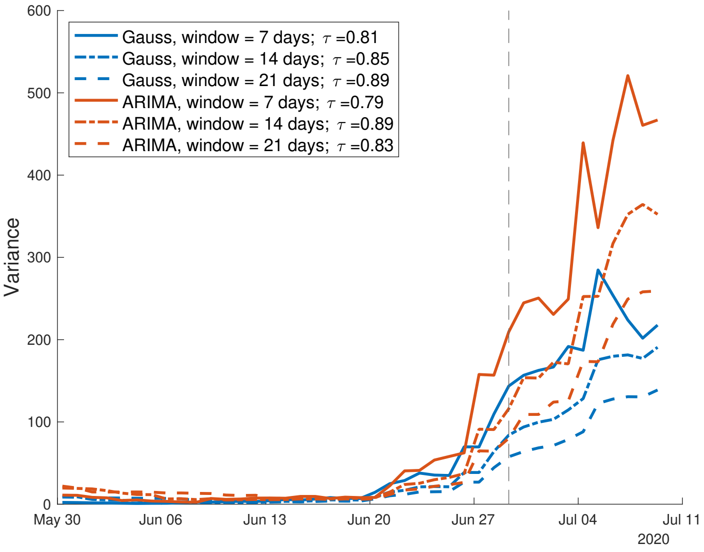

Fig I. Analysis of the variance in the Luxembourg setting. Its increase is evident prior to the transition (dashed vertical line). τ, which quantifies the overall increasing trend, is little sensitive to the sliding window size, as displayed by the three curves and by τ values reported in the text. The variance is computed over the residuals from Gaussian filtering and ARIMA detrending. The increasing trend during the considered time window is quantified by the associated τ values.

speculate that the detection performance might be linked to the sensitivity of the skewness to the noise distribution (as already introduced in [37]): potentially, the correct combination of fluctuations and approach to the $R(t) = 1$ might have yielded the observed result. Here, we limit ourselves to reporting the observation, demanding further analytical and computational studies to investigate the issue. In any case, the skewnees is again no better than a random classifier on the $\mathcal{N}$ set.

	Gaussian det.		ARIMA det.	
Indicator	over $\mathcal{Y}$	over $\mathcal{N}$	over $\mathcal{Y}$	over $\mathcal{N}$
Variance	0.6718	0.4681	0.7334	0.2561
AC(1)	0.5234	0.3132	0.2624	0.6021
CV	0.3370	0.2019	0.4380	0.1813
Skewness	0.7826	0.5005	0.6805	0.4991

Table C. AUC scores for different indicators, over $\mathcal{Y}$ and $\mathcal{N}$ datasets, after Gaussian or ARIMA detrending methods.

Overall, this complementing analysis on incidence data provides additional insights and questions, to be further compared with theoretical studies. Nonetheless, it contributes in stressing one of the main points of the main study: as the considered indicators rely on a number of assumptions, we are justified in using them when such assumption are satisfied, which is not always the case in real-world settings. Otherwise, the EWS sensitivity to such assumptions might yield spurious signals and hinder our capability to extend them in uncertain contexts.

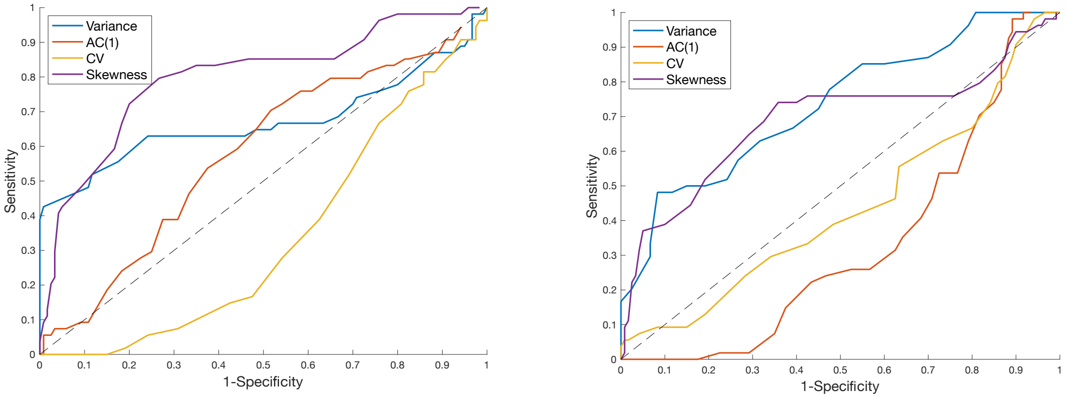

Fig J. ROC curves for each considered indicator, with sensitivity and specificity calculated on each timepoint for all countries in $\mathcal{Y}$. Each point corresponds to a test value for τ, to define if the detection if positive. The diagonal line corresponds to the ROC of a random classifier. Curves above it imply better performance. Left: Computed on Gaussian filtered data. Right: Computed on ARIMA detrended data.

Country	ARIMA parameters	Residuals mean	Residuals std
State of Victoria (Australia)	(2,1,0)	0.39	52.82
Austria	(1,1,4)	5.43	93.63
Denmark	(3,1,0)	3.17	46.87
Israel	(0,1,1)	0.41	349.6
Japan	(7,1,5)	3.58	110.0
Korea, South	(0,1,1)	0.501	58.96
Luxembourg	(2,1,5)	0.319	25.92
Nepal	(0,1,1)	-0.099	91.999
Singapore	(1,1,1)	0.46	121.61
Veneto (Italy)	(5,1,3)	1.25	50.66

Table D. ARIMA model parameter combinations over incidence data, residuals mean and standard deviation, for each country.

References

1. Anderson RM, May RM. Population biology of infectious diseases: Part I. Nature. 1979;280(5721):361–367.

2. Proverbio D, Kemp F, Magni S, Husch A, Aalto A, Mombaerts L, et al. Dynamical SPQEIR model assesses the effectiveness of non-pharmaceutical interventions against COVID-19 epidemic outbreaks. PloS one. 2021;16(5):e0252019.

3. O'Regan SM, Drake JM. Theory of early warning signals of disease emergenceand leading indicators of elimination. Theoretical Ecology. 2013;6(3):333–357.

4. Wu JT, Leung K, Leung GM. Nowcasting and forecasting the potential domestic and international spread of the 2019-nCoV outbreak originating in Wuhan, China: a modelling study. The Lancet. 2020;395(10225):689–697.

5. Peng L, Yang W, Zhang D, Zhuge C, Hong L. Epidemic analysis of COVID-19 in China by dynamical modeling. arXiv. 2020;preprint arXiv:2002.06563.

6. Liu Y, Gayle AA, Wilder-Smith A, Rocklöv J. The reproductive number of COVID-19 is higher compared to SARS coronavirus. Journal of Travel Medicine. 2020;27(2).

7. Berglund N, Gentz B. Noise-induced phenomena in slow-fast dynamical systems: a sample-paths approach. Springer Science & Business Media; 2006.

8. Ashwin P, Wieczorek S, Vitolo R, Cox P. Tipping points in open systems: bifurcation, noise-induced and rate-dependent examples in the climate system. Philosophical Transactions of the Royal Society A. 2012;370(1962):1166–1184.

9. Flaxman S, Mishra S, Gandy A, Unwin HJT, Mellan TA, Coupland H, et al. Estimating the effects of non-pharmaceutical interventions on COVID-19 in Europe. Nature. 2020;584(7820):257–261.

10. Alonso D, McKane AJ, Pascual M. Stochastic amplification in epidemics. Journal of the Royal Society Interface. 2007;4(14):575–582.

11. Southall E, Tildesley M, Dyson L. Prospects for detecting early warning signals in discrete event sequence data: Application to epidemiological incidence data. PLoS Comput Biol. 2020;16(9):e1007836.

12. Van Kampen NG. Stochastic processes in physics and chemistry. Elsevier; 1992.

13. Dakos V, Scheffer M, van Nes EH, Brovkin V, Petoukhov V, Held H. Slowing down as an early warning signal for abrupt climate change. Proceedings of the National Academy of Sciences. 2008;105(38):14308–14312.

14. Boettiger C, Hastings A. Early warning signals and the prosecutor's fallacy. Proceedings of the Royal Society B. 2012;279(1748):4734–4739.

15. Scheffer M, Carpenter SR, Lenton TM, Bascompte J, Brock W, Dakos V, et al. Anticipating critical transitions. science. 2012;338(6105):344–348.

16. Kuznetsov YA. Elements of applied bifurcation theory. vol. 112. Springer Science & Business Media; 2013.

17. Lee J. Introduction to topological manifolds. vol. 202. Springer Science & Business Media; 2010.

18. Strogatz SH. Nonlinear dynamics and chaos with student solutions manual: With applications to physics, biology, chemistry, and engineering. CRC press; 2018.

19. Kuehn C. A mathematical framework for critical transitions: Bifurcations, fast–slow systems and stochastic dynamics. Physica D: Nonlinear Phenomena. 2011;240(12):1020–1035.

20. Allen LJ. An introduction to stochastic epidemic models. In: Mathematical epidemiology. Springer; 2008. p. 81–130.

21. O'Regan SM, Burton DL. How stochasticity influences leading indicators of critical transitions. Bulletin of mathematical biology. 2018;80(6):1630–1654.

22. Reno C, Lenzi J, Navarra A, Barelli E, Gori D, Lanza A, et al. Forecasting COVID-19-Associated Hospitalizations under Different Levels of Social Distancing in Lombardy and Emilia-Romagna, Northern Italy: Results from an Extended SEIR Compartmental Model. Journal of clinical medicine. 2020;9:1492.

23. Reiner RC, Barber RM, Collins JK, Zheng P, Adolph C, Albright J, et al. Modeling COVID-19 scenarios for the United States. Nature Medicine. 2020;27:94–105.

24. Kemp F, Proverbio D, Aalto A, Mombaerts L, d'Herouel AF, Husch A, et al. Modelling COVID-19 dynamics and potential for herd immunity by vaccination in Austria, Luxembourg and Sweden. Journal of Theoretical Biology. 2021; p. 110874.

25. Dong E, Du H, Gardner L. An interactive web-based dashboard to track COVID-19 in real time. The Lancet infectious diseases. 2020;20(5):533–534.

26. Roser M, Ritchie H, Ortiz-Ospina E, Hasell J. Coronavirus Pandemic (COVID-19). Our World in Data. 2020;https://ourworldindata.org/coronavirus.

27. Abbott S, Hellewell J, Munday J, Chun J, Thompson R, Bosse N, et al. Temporal variation in transmission during the COVID-19 outbreak [version 1; peer review: awaiting peer review]. Wellcome Open Res. 2020;5(112).

28. Brett T, Ajelli M, Liu QH, Krauland MG, Grefenstette JJ, van Panhuis WG, et al. Detecting critical slowing down in high-dimensional epidemiological systems. PLoS computational biology. 2020;16(3):e1007679.

29. Ajelli M, Merler S, Fumanelli L, y Piontti AP, Dean NE, Longini IM, et al. Spatiotemporal dynamics of the Ebola epidemic in Guinea and implications for vaccination and disease elimination: a computational modeling analysis. BMC medicine. 2016;14(1):1–10.

30. Liu QH, Ajelli M, Aleta A, Merler S, Moreno Y, Vespignani A. Measurability of the epidemic reproduction number in data-driven contact networks. Proceedings of the National Academy of Sciences. 2018;115(50):12680–12685.

31. Bettencourt LM, Ribeiro RM. Real time bayesian estimation of the epidemic potential of emerging infectious diseases. PloS one. 2008;3(5):e2185.

32. Du Z, Xu X, Wu Y, Wang L, Cowling BJ, Meyers LA. Serial interval of COVID-19 among publicly reported confirmed cases. Emerging infectious diseases. 2020;26(6):1341.

33. Park M, Cook AR, Lim JT, Sun Y, Dickens BL. A systematic review of COVID-19 epidemiology based on current evidence. Journal of clinical medicine. 2020;9(4):967.

34. Benvenuto D, Giovanetti M, Vassallo L, Angeletti S, Ciccozzi M. Application of the ARIMA model on the COVID-2019 epidemic dataset. Data in brief. 2020;29:105340.

35. O'Dea EB, Drake JM. Disentangling reporting and disease transmission. Theoretical Ecology. 2019;12(1):89–98.

36. Wilmes P, Zimmer J, Schulz J, Glod F, Veiber L, Mombaerts L, et al. SARS-CoV-2 transmission risk from asymptomatic carriers: results from a mass screening programme in Luxembourg. Lancet Reg Heal-Europe. 2021;4:100056.

37. Guttal V, Jayaprakash C. Changing skewness: an early warning signal of regime shifts in ecosystems. Ecology letters. 2008;11(5):450–460.

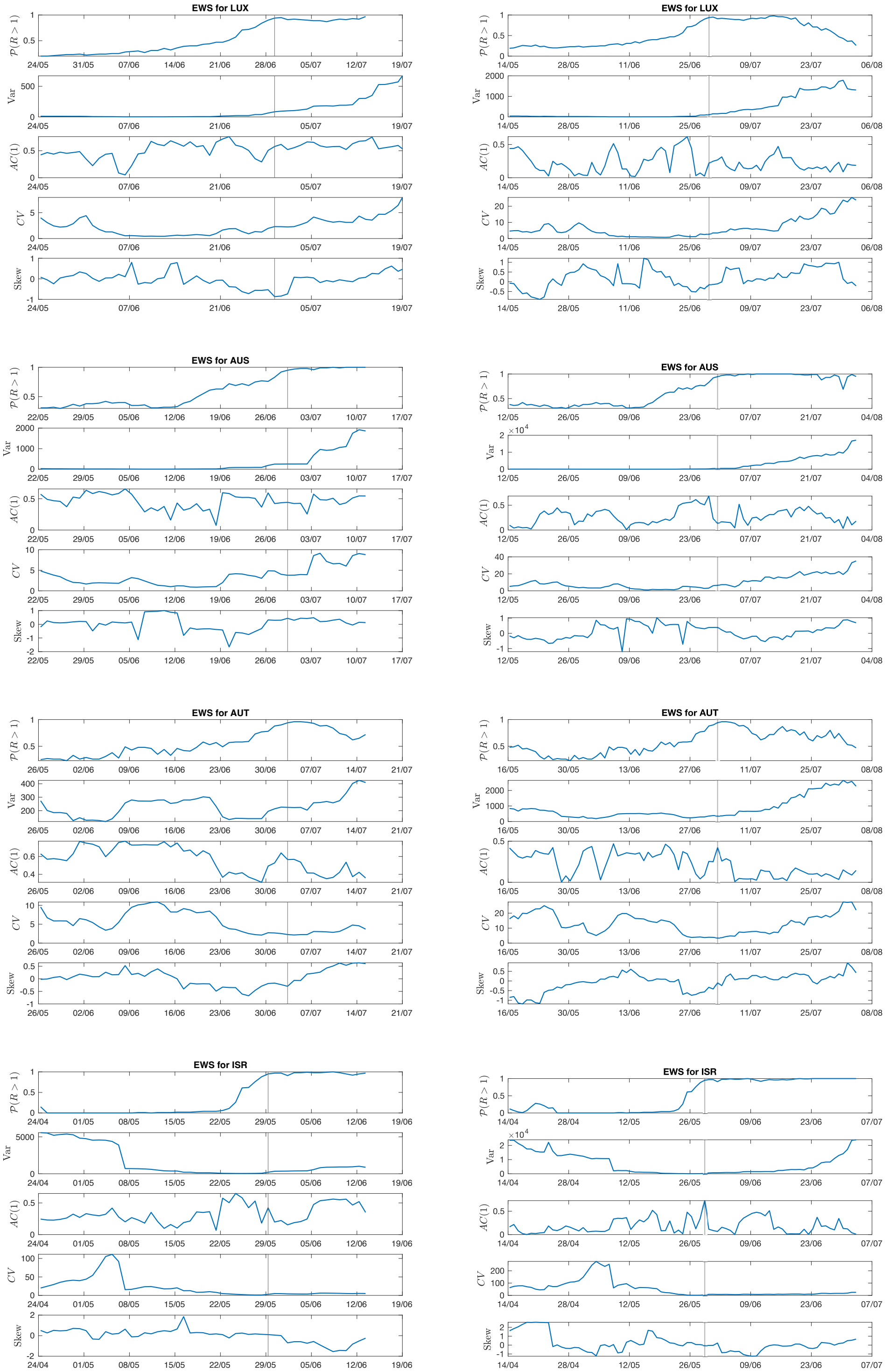

Fig K. Evolution of EWS far from the transition point. Four example countries are shown: Luxembourg and Austria, with controlled features; State of Victoria (Australia), with small deviations from controlled features; and Israel that does not satisfy theoretical conditions. Considered EWS are the most common ones (variance, lag-1 autocorrelation, coefficient of variation, skewness). In addition, to mark the approach to the transition, $\mathcal{P}(R(t) > 1)$ from the Bayesian estimation (see Main Text) is displayed. The vertical line reports the transition date. Left column: detrending method employed: Gaussian filtering. Right column: detrending method employed: ARIMA.
