# Supplementary material for: Performance of early warning signals for disease re-emergence: A case study on COVID-19 data
Source: PLoS Comput Biol. 2022 Mar 30;18(3):e1009958. doi: 10.1371/journal.pcbi.1009958 (PMC9000113; doi:10.1371/journal.pcbi.1009958)
Supplement: Attachment — Submitted filename: REVIEW Performance of EWS for disease re-emergence_ a case study on COVID-19 data.docx [file pcbi.1009958.s002.docx]

This paper presents an empirical study of EWSs of disease re-emergence, using collected COVID-19 data at the country-level. The main focus of the study is to understand if characteristics of EWS can be identified in some countries which underwent a second COVID wave. The study discussed the reasoning why some EWS may not be present in some countries, claiming that they failed to detect traits of critical slowing down for cases with rapid re-emergence or cases with fat-tailed noise in the data. Proverbio et al., calculate the rate of emergence and the noise in the data for different countries, before calculating EWSs - to see if the impact of these two traits will lead to different time-changing behaviours in EWSs. As highlighted by the authors, there are few empirical studies on EWSs of disease transitions and I think this paper contributes to this gap in the literature nicely. However, I think the conclusions of the paper are slightly overreaching. Particularly the claim that EWSs will fail to work for diseases with rapid emergence or for fat-tailed noise. Without further theoretical studies, I don’t think this claim is rigorously supported in this paper and instead I think this needs to be made clear in the abstract (and paper) that this claim is instead considered here as a contributing factor.

**Major**

1. On line 286 (page 8), it describes countries with a “faster evolution” of emergence and that this does not satisfy the assumption of slow evolution. Could the authors comment on how they determine what rate of emergence is too fast (what the cut off is). Is there a parameter region of emergence rates which are appropriate?
2. On line 294-302 it is unclear how each country is chosen as set Y or set N. A table would be beneficial for the reader, with headings of each country and containing information on: the population size, country size (e.g. km^2), covid restrictions during the period considered, average number of tests per week and a summary of your figures 1b and 1c. This information is included for Luxembourg (lines 297-293), but it would be useful to have the same summary information for all countries. In particular, it is not very obvious why some countries are placed into the N category.

Is your analysis on when R(t) crosses through 1 used in the selection of sets N and Y? Have you considered how sensitive the emergence rates and noise are to the time period selected from the R(t) analysis and would this change which countries are selected?

3. The calculation of the ROC curves is not clear (lines 251 to 265). Are you taking the Kendall’s tau score over windows before the critical transition as your positive data set and Kendall’s tau score after the critical transition as your negative data set? This would then attempt to classify which Kendall’s tau scores are pre- or post- transition, but I’m not sure if this analysis is appropriate. If you consider the analytical behaviour of variance prior to disease emergence (see O’Dea et al., 2019 or Brett et al. 2018), the variance is expected to increase both before and after the critical transition. This would give a Kendall’s tau score near 1 before the transition and a score near 1 after the transition.
4. For the data curation, are you considering positive PCR tests (line 146) - i.e. is the test procedure consistent across the different countries and have you accounted for the sensitivity and specificity of these tests? Please clearly state what you mean by active cases - is this a daily random sample (i.e. including all ages and asymptomatics) of the entire population to find the proportion of the population which is infected?
5. In your proxy to active cases (equation 4), does this account for the number of tests taken? How are the number of recovered patients measured and is this a publicly available dataset? Please be specific about which country had active screening available and which countries used this proxy.
6. I think it would be appropriate to repeat this analysis but instead use the proportion of positive tests for each country (rather than your proxy). The analytical behaviour of EWSs calculated on incidence-type data has previously been considered (O’Dea et al and Southall et al); and for disease emergence EWSs behave similarly to prevalence type data. I think this would be more suitable than using a proxy for prevalence which can introduce additional errors/limitations.
7. Bifurcation delays are discussed on line 250, 366-367 and 375; however this may not be a bifurcation lag but instead a confounding factor of lags within the data. In particular, please discuss the impact of the latency period for COVID (e.g. average of 5 days until symptoms appear) and the impact of reporting delays (e.g. PCR tests take on average 24 hours) - which could also introduce lags in the data and the results.

**Minor**

1. In the abstract you discuss “active monitoring” and online detection is also discussed on line 369. In the paper, online/real-time detection is not used and so I think this should be made clear that it is future work.
2. Figure 4, please include the plots of the ARIMA detrending for each country. Also please set the y axis to be the same for all countries. In particular, in Israel, it does look like variance increases prior to the critical transition, but this behaviour is very small as the yscale is set to cover dynamics after the critical transition.
3. Discussion please discuss the limitations of your study. Including the data curation, calculation of R(t), quantity of data, etc
4. Introduction: the following papers also contain empirical studies of EWS on disease transitions and should be included. What are the limiting features of these empirical studies and how might your new study contribute to reducing these limitations?
   a. Harris, M. J., Hay, S. I., & Drake, J. M. (2020). Early warning signals of malaria resurgence in Kericho, Kenya. Biology letters, 16(3), 20190713.
   b. Brett, T. S., & Rohani, P. (2020). Dynamical footprints enable detection of disease emergence. PLoS biology, 18(5), e3000697.
   c. Kaur, T., Sarkar, S., Chowdhury, S., Sinha, S. K., Jolly, M. K., & Dutta, P. S. (2020). Anticipating the novel coronavirus disease (COVID-19) pandemic. Frontiers in public health, 8, 521.
   d. Liu, R., Zhong, J., Hong, R., Chen, E., Aihara, K., Chen, P., & Chen, L. (2021). Predicting local COVID-19 outbreaks and infectious disease epidemics based on landscape network entropy. Science Bulletin.
   e. Southall, E., Brett, T. S., Tildesley, M. J., & Dyson, L. (2021). Early warning signals of infectious disease transitions: a review. Journal of the Royal Society Interface, 18(182), 20210555.

Please also comment further on how this study compares to the other published empirical study of EWSs of COVID-19 emergence (Kaur et al. and Liu et al.).

5. Introduction: the effective reproduction number (or the time-varying reproduction number R(t)), which is what you calculate, is not the same as the basic reproduction number R_0 which relates to the transcritical bifurcation point. Please introduce both and highlight their differences.
6. Gaussian detrending: what bandwidth (standard deviation of the Gaussian filter) was taken? Depending on the bandwidth of the Gaussian filter, it is not surprising that the Gaussian filter is highly correlated to the linear moving average filter (on line 315). Also line 194, Dessavre et al., don’t recommend Gaussian detrending (citation [46]) In fact, they found that EWSs can exhibit odd behaviours when a moving window method is used to detrend the data.
7. Can table 2 be written as |AUC - 0.5|. As you pointed out, an AUC score of 0 means that it can be perfectly classified by its negative trend, so CV and variance seem to be classified very well for the N data set - could you discuss what this might tell us.
8. AC(1) is very noisy and using a short time series is not recommended
9. In figure 1b, can the excess kurtosis be calculated instead (Kurt - 3). This would make it easier to read this figure.
