# Supplementary material for: Performance of early warning signals for disease re-emergence: A case study on COVID-19 data
Source: PLoS Comput Biol. 2022 Mar 30;18(3):e1009958. doi: 10.1371/journal.pcbi.1009958 (PMC9000113; doi:10.1371/journal.pcbi.1009958)
Supplement: Attachment — Submitted filename: PLoS_Computational_biology_COVID_EWS_review.docx [file pcbi.1009958.s003.docx]

The authors use recent COVID-19 data from multiple countries to perform a careful analysis of the performance of EWS at detecting resurgent outbreaks. As the authors state, there has been limited studies attempting to quantify EWS performance using actual epidemiological data (rather than simulated data).

I enjoyed reading the paper, and found the overall study was carefully designed and well motivated. I found the paper well-suited to PLoS Computational Biology, however there were some points in the methods I remain unclear on and I do have some concerns with some decisions made in the analysis.

Provided these issues are addressed I would be willing to recommend accepting the paper.

Major issues:

Data:

The authors calculate the EWS from the number of “active cases” in the population. Active cases are calculated from epidemiological data as the difference between cumulative confirmed cases and cumulative deaths plus confirmed recovered (Eq. 4). However, my experience of COVID-19 data is that the “recovered cases” dataset is usually highly incomplete: Many individuals test positive then recover at home with no further medical system contact. Using Eq. 4, these missing recoveries correspond to an infinite infectious period — i.e. the error in estimated active cases will accumulate over time and may substantially differ from the number of active cases. At an extreme, this accumulating error might give the illusion of re-emergence in locations where it might not have occurred. Unless the authors are confident that robust public health protocols are in place for following up on the infectious status of all confirmed cases for every country studied, I strongly advise not calculating active cases in this way.

I appreciate the issue the authors are trying to resolve: That most of the theory is for prevalence data (as the authors explain in the supplementary material). However I’m worried the active cases approach introduces more problems than it solves. If missing recoveries are an issue I suggest calculating the EWS using incidence data (such as daily confirmed cases), see Refs. 15 and 16 for details.

Bayesian inference:

I’m confused how the Bayesian inference was performed (after reading both main manuscript supplementary information), and think the section in the main manuscript needs to be a lot more comprehensive. E.g. was a rolling window (from *t* to *T*) used? Was the window centred? What MCMC algorithm was used? Also how exactly was $t_{em}$ determined? Its unclear to me whether it was the time of the first maximum of $P(R(t)>1)$ or whether when $P(R(t)>1)=1$.

Timeseries detrending:

While the estimator of the variance (Eq. 7) is careful to ensure that the variance at time *t*, ${Var}_{i,t}$, only depends on data up to time *t*, i.e. it is non-anticipating, I’m unclear if this is also the case for all the detrending methods (e.g. ARIMA and Gaussian kernel). If removing the trend at time *t* relies on data “from the future” then it becomes hard to assess EWS performance at detecting emergence.

AOC analysis:

Lines 251-256 left me unclear how exactly the ROC curve was calculated, i.e. exactly which data (both time range and which countries) were used to calculate Kendall’s tau for the test and null cases. One issue identified in Ref. 18 is that an initial downward trend in incidence (before resurgence) can mask the ability of EWS to detect resurgence. Was this an issue for any of the countries studied (it’s hard to know without details on what dates were used for EWS calculation and Kendall’s tau calculations)?

Figures:

Figure 1: State which country the example time series (panel a) corresponds to.

Figure 2: Formatting error of first sentence. State which time series is being detrended and shown in panels a b and c (is in Luxembourg and the same as Figure 1a?). I’m surprised the ARIMA and Gaussian are so poorly correlated. Do you have any explanation why? Do you know why the residuals around the centre are so much smaller than the others? Also it’s hard to relate the x axis (date points) to the x axis of other figures — maybe stick to date like Figure 1 and 3

Figure 5: The caption confused me (for the same reasons as the section on the ROC and needs reworking). I also don’t understand how the curves could represent averages over counties in the test set? Are the value of tau for each country not the samples used in calculating the ROC curve?

Minor issues:

Line 27: Sentence unclear: “If this would be the case, the consequences would be far-reaching, possibly expanding the set of epidemic indicators, using complexity theory.”

Line 141: Type: “based on the theory recalled above theory and”

Lines 151-175: I’m not sure on the journals style but if possible it would be better to format data sources as reference rather than URLs in the main text.

Line 167: Typo in “country-wide”
