# Supplementary material for: Performance of early warning signals for disease re-emergence: A case study on COVID-19 data
Source: PLoS Comput Biol. 2022 Mar 30;18(3):e1009958. doi: 10.1371/journal.pcbi.1009958 (PMC9000113; doi:10.1371/journal.pcbi.1009958)
Supplement: Attachment — Submitted filename: Performance-of_response-reviewers.docx [file pcbi.1009958.s004.docx]

Dear editor, dear reviewers,

We thank you for your careful review and for the list of suggested improvements. Please find below detailed responses to the points you raised. Our responses are in blue. In the main text, the parts we edited, following your suggestions, are in red. Moreover, Fig. 1, 2 and 4 have been edited, including the reviewers’ suggestions for better visualization clarity.

The Supplementary Material has also been extended to include the reviewers’ suggestions and to expand the analysis presented in the main text; in particular, we have included Table S1 to report other useful information about the considered countries, an additional section (Sec.S7) to further discuss AUC values, and an additional section (Sec. S8) focusing on incidence data.

**Reviewer #1**

This paper presents an empirical study of EWSs of disease re-emergence, using collected COVID-19 data at the country-level. The main focus of the study is to understand if characteristics of EWS can be identified in some countries which underwent a second COVID wave. The study discussed the reasoning why some EWS may not be present in some countries, claiming that they failed to detect traits of critical slowing down for cases with rapid re-emergence or cases with fat-tailed noise in the data. Proverbio et al., calculate the rate of emergence and the noise in the data for different countries, before calculating EWSs - to see if the impact of these two traits will lead to different time-changing behaviours in EWSs. As highlighted by the authors, there are few empirical studies on EWSs of disease transitions and I think this paper contributes to this gap in the literature nicely. However, I think the conclusions of the paper are slightly overreaching. Particularly the claim that EWSs will fail to work for diseases with rapid emergence or for fat-tailed noise. Without further theoretical studies, I don’t think this claim is rigorously supported in this paper and instead I think this needs to be made clear in the abstract (and paper) that this claim is instead considered here as a contributing factor.

We are happy to read Reviewer #1’s positive assessment and we thank him/her for the useful suggestion about how to rephrase our contribution in a balanced manner. We edited the tone and message of abstract and main text (throughout the text) accordingly.

**Major**

1. On line 286 (page 8), it describes countries with a “faster evolution” of emergence and that this does not satisfy the assumption of slow evolution. Could the authors comment on how they determine what rate of emergence is too fast (what the cut off is). Is there a parameter region of emergence rates which are appropriate?
   Since it is difficult to avoid an arbitrary bias in setting a hard threshold to reproduce the mathematical limit epsilon << 1 (from Eq. 1), we have used group clustering as a modelling assumption. From the results shown in Fig. 1b,c, State of Victoria, Austria, Luxembourg, Singapore and Veneto are grouped together to represent the assumption of slow rate. Moreover, except for Australia, their noise distribution is close to Gaussian, as can be seen from the corresponding values of skewness and kurtosis in Fig. 1b. They thus form the test set Y, used to further assess the performance of EWS. On the other hand, Denmark, Israel and Nepal display higher rates of approach to R(t)=1 and large deviations for Gaussian noise distribution. Hence, they are grouped together in a set N; this is used to interpret the performance of EWS in settings that are not properly described by theoretical models and represent possible limitations of the predetermined predictions. South Korea and Japan are more ambiguous when clustering over the slope of R(t), therefore we split them into Y and N, respectively, based on their relative vicinity to Gaussian noise distribution.
   This way, we have subdivided the considered countries into two test sets, based on whether the country is representative or not of the satisfaction of some modelling assumptions (slow approach to the transition and noise distribution close to white.)
   In order to make our explanation clearer, we have edited the text in lines 331-346.
2. On line 294-302 it is unclear how each country is chosen as set Y or set N. A table would be beneficial for the reader, with headings of each country and containing information on: the population size, country size (e.g. km^2), covid restrictions during the period considered, average number of tests per week and a summary of your figures 1b and 1c. This information is included for Luxembourg (lines 297-293), but it would be useful to have the same summary information for all countries. In particular, it is not very obvious why some countries are placed into the N category. Is your analysis on when R(t) crosses through 1 used in the selection of sets N and Y?

The sets Y and N were chosen according to the observed features of noise distribution and rate of approach to R(t)=1 (not the time when R(t) crosses 1). We also refer to the answer above for further details. Other information about population size and COVID restrictions were only considered for Luxembourg to specify why we consider it a controlled sample for the careful analysis of the EWS next to the transition, while the other countries were subdivided solely on the basis of the dynamical features. Hence, we opted to not include the suggested table in the main text, to avoid any potential source of confusion. We have nonetheless included it into the Supplementary Material, as it nicely complements the additional explanation about data curation (refer to Section S2 and Table S1). Since we acknowledge that our original explanation could have been confusing, we have edited the text in lines 331-346.
Have you considered how sensitive the emergence rates and noise are to the time period selected from the R(t) analysis and would this change which countries are selected?
We have tested the sensitivity to the time period selected. For some countries (e.g. Nepal and Israel in Fig. S4) the choice of time period is rather unique, in order to provide a good linear fit. When the choice could be more flexible, e.g. for Luxembourg or Veneto, we have made sure that changes in the time period up to ±15 days are well represented by the confidence intervals of the estimated and reported slope of R(t) (cf. Sup Mat Fig. S4).

3. The calculation of the ROC curves is not clear (lines 251 to 265). Are you taking the Kendall’s tau score over windows before the critical transition as your positive data set and Kendall’s tau score after the critical transition as your negative data set? This would then attempt to classify which Kendall’s tau scores are pre- or post- transition, but I’m not sure if this analysis is appropriate. If you consider the analytical behaviour of variance prior to disease emergence (see O’Dea et al., 2019 or Brett et al. 2018), the variance is expected to increase both before and after the critical transition. This would give a Kendall’s tau score near 1 before the transition and a score near 1 after the transition.

We thank Reviewer #1 for this opportunity to make our text clearer. First, we compute each indicator over a rolling window of 14 days, starting from 30 days before the transition (long enough to have several data points, but not too long to overlap with the first wave) up to 5 days after the transition (to account for possible small bifurcation delays). These were the test cases. For the null cases, we use τ values taken long before the transition occurs, that is on windows associated with timepoints t<-30, i.e. more than 30 days before the transition. We compare each statistical indicator’s ability to correctly distinguish which Kendall's tau scores belong to those from before or after re-emergence, that is, we determine whether the estimated τ is higher or lower than some threshold value at that timepoint and determine whether each simulation is classified correctly by that threshold. This gives a proportion of true positives and false positives. To do so, we compare various values from 0<τ<1 to those of the positive and negative data set, for each country. We calculate the indicator for each country in a test set at the given timepoint, and then group the specificity and sensitivity results to obtain the final ROC curve. We have now edited the text to incorporate this extended explanation in lines 291-300 of Sec. 2.5.

4. For the data curation, are you considering positive PCR tests (line 146) - i.e. is the test procedure consistent across the different countries and have you accounted for the sensitivity and specificity of these tests? Please clearly state what you mean by active cases - is this a daily random sample (i.e. including all ages and asymptomatics) of the entire population to find the proportion of the population which is infected?

We are considering all officially and publicly available reported COVID-19 cases. They have been mostly confirmed by positive PCR tests but further details about specificity and sensitivity are not disclosed for all countries. For Luxembourg, active cases are reported by the Ministry of Health among other statistics and they derive from random samples over the whole population, following a large-scale testing strategy (Wilmes et al., 2021). This has now been clarified in the text (lines 160-165).

5. In your proxy to active cases (equation 4), does this account for the number of tests taken? How are the number of recovered patients measured and is this a publicly available dataset? Please be specific about which country had active screening available and which countries used this proxy.

We use publicly available data from official sources listed in the text. We acknowledge that estimates of recovered cases may be biased due to their limited tracking, and no disclosure about how recovered patients are specifically counted is provided in the sources. However, we have tried to best limit the biases associated with the used data. As described in Sec. 2.2, we curated the dataset following several quality criteria. We did not consider all countries displaying clear deviations from expected epidemic trends in their proxy *A* (e.g. “saw-like” curves or not decreasing curves like the ones displayed in Fig. S2); during the data analysis, these were in most parts associated with biased reported recovered cases, for instance reported all in one day. We did not consider those countries with a share of positive tests > 5% close to the transition, to limit as much as possible the effects of undertesting (the cutoff has been chosen in accordance with WHO guidelines and is how we account for the number of tests taken). We also did not consider regional areas where the assumptions of homogenous mid-field diffusion clearly do not hold (e.g., where spacial heterogeneity is recognized in the literature, or when there were very few cases over the whole considered time period, mostly representative of stochastic fluctuations).

Luxembourg is a different case: since the country is small and the hospital system was carefully monitored, we are more confident about the numbers reported. As a consequence, we have first concentrated on this country for the initial comparisons of empirical analysis and theoretical predictions.

6. I think it would be appropriate to repeat this analysis but instead use the proportion of positive tests for each country (rather than your proxy). The analytical behaviour of EWSs calculated on incidence-type data has previously been considered (O’Dea et al and Southall et al); and for disease emergence EWSs behave similarly to prevalence type data. I think this would be more suitable than using a proxy for prevalence which can introduce additional errors/limitations.
   We initially employ prevalence data, because most of the theoretical results in literature are derived for this quantity, rather than for incidence. In order to compare our results to several literature studies (including those specifically dedicated to critical transitions in general), we have first focused on this aspect. Nonetheless, we agree with Reviewer #1 that more recent studies have considered the expected behavior of incidence data, and that, despite our efforts to minimize the impact of biases in our proxy for *A,* we cannot rule them out completely. Regarding the issue of testing, we were careful in constructing our dataset using only countries for which the share of positive tests was <5% (to minimize the chance of undertesting, as per WHO guidelines.) Hence, we followed Reviewer #1’s suggestion to repeat the analysis on incidence data. It is now included and discussed in the text (lines 192-200 and 579-597) and all the corresponding results have been included in electronic supplementary information Sec S8.
7. Bifurcation delays are discussed on line 250, 366-367 and 375; however this may not be a bifurcation lag but instead a confounding factor of lags within the data. In particular, please discuss the impact of the latency period for COVID (e.g. average of 5 days until symptoms appear) and the impact of reporting delays (e.g. PCR tests take on average 24 hours) - which could also introduce lags in the data and the results.
   We agree on Reviewer #1’s point. If the mentioned sources of delay are constant and constantly distributed, then the bifurcation delay would be the primary explanation for the observed lags. Unfortunately, it is difficult to determine the real impact of latency periods and reporting delays on the transition delays; for this, we would need additional data to perform a nowcasting study. We have included this discussion in lines 424-427 and lines 570-574.

**Minor**

1. In the abstract you discuss “active monitoring” and online detection is also discussed on line 369. In the paper, online/real-time detection is not used and so I think this should be made clear that it is future work.

In light of Reviewer #1’s observation we would like to clarify our approach. To calculate the residuals and all the estimated EWS, we have employed sliding windows that are always associated with the rightmost point. This way, the estimates are agnostic of future values and only become aware of them as they are available. In this way, we mimic what occurs in real-time online monitoring, i.e., the estimation of an indicator as soon as a new data point becomes available. We have clarified this point in lines 257-259.

2. Figure 4, please include the plots of the ARIMA detrending for each country.
   This is a nice suggestion, to allow readers to better interpret the effect of both detrending methods. We have thus included the plots in Fig. 4 and expanded our discussion in lines 411-413 and 438-445.
   Also please set the y axis to be the same for all countries. In particular, in Israel, it does look like variance increases prior to the critical transition, but this behaviour is very small as the yscale is set to cover dynamics after the critical transition.
   We indeed agree with the reviewer that we can improve this figure’s readability. Instead of setting the y-scale equal to all countries (which would cut off some higher variance values), we have zoomed a bit more in the vicinity of the transition, neglecting what happens more than one month after that. This way, the behavior next to the transition is properly magnified.
3. Discussion please discuss the limitations of your study. Including the data curation, calculation of R(t), quantity of data, etc

The discussion of the limitations of this study has been extended, better reflecting the remarks on data quality, the robustness of the estimators and of the methods to calculate them (lines 556-577).

4. Introduction: the following papers also contain empirical studies of EWS on disease transitions and should be included. What are the limiting features of these empirical studies and how might your new study contribute to reducing these limitations?
   a. Harris, M. J., Hay, S. I., & Drake, J. M. (2020). Early warning signals of malaria resurgence in Kericho, Kenya. Biology letters, 16(3), 20190713.
   b. Brett, T. S., & Rohani, P. (2020). Dynamical footprints enable detection of disease emergence. PLoS biology, 18(5), e3000697.
   c. Kaur, T., Sarkar, S., Chowdhury, S., Sinha, S. K., Jolly, M. K., & Dutta, P. S. (2020). Anticipating the novel coronavirus disease (COVID-19) pandemic. Frontiers in public health, 8, 521.
   d. Liu, R., Zhong, J., Hong, R., Chen, E., Aihara, K., Chen, P., & Chen, L. (2021). Predicting local COVID-19 outbreaks and infectious disease epidemics based on landscape network entropy. Science Bulletin.
   e. Southall, E., Brett, T. S., Tildesley, M. J., & Dyson, L. (2021). Early warning signals of infectious disease transitions: a review. Journal of the Royal Society Interface, 18(182), 20210555.

Please also comment further on how this study compares to the other published empirical study of EWSs of COVID-19 emergence (Kaur et al. and Liu et al.).
We thank Reviewer #1 for his/her suggestions about further literature that contributes to expanding our introduction. The relevant references (a., c., e.; ref b. was already included) has now been included and briefly discussed in the text (lines 33-38). Although potentially relevant to cover the recent literature on EWS for epidemics, we have not included Kaur et al. (2020). In fact, it focuses on the first COVID-19 wave, potentially suffering from undertesting. In addition, the emergence of COVID-19 is not a zero-eigenvalue bifurcation, but is a novelty followed by exponential growth. As the epidemic takes on (and not before), the variance rises since the mean rises. Therefore, we did not consider the first wave, but only subsequent re-emergences after R(t) had dropped <1. Therefore, it is difficult to make a direct comparison with our study. Regarding Liu et al. (2021), we acknowledge its relevance for practical applications in epidemic monitoring, but it is slightly complicated to compare with our results: in that article, the authors employ a special indicator deriving from considerations on critical transitions, but not analytically tractable and for which no prior literature on epidemics was available.

5. Introduction: the effective reproduction number (or the time-varying reproduction number R(t)), which is what you calculate, is not the same as the basic reproduction number R_0 which relates to the transcritical bifurcation point. Please introduce both and highlight their differences.

Reviewer #1 suggests distinguishing between the static representation of the transcritical bifurcation for every steady-state value of R and the dynamical crossing of the bifurcation happening when R is allowed to change in a quasi-steady-state manner (R(t)). We thank him/her for pointing out the importance of stressing this distinction. Indeed, the basic reproduction number R_0 represents the leading parameter driving the spread of an epidemic, provided that no changes are observed in the original transmission dynamics. As epidemic parameters might change over time, e.g. reflecting the effect of pharmaceutical or non-pharmaceutical interventions, or since the pool of susceptible individuals shrinks with time due to people getting infected, we speak of effective time-varying reproduction number R = R_0 * f(t), where f(t) is a time-dependent function. Hence, in a non-steady-state setting, the time dependency guides the reproduction number over the bifurcation point, which otherwise would not be crossed. We have now introduced this conceptual distinction in the text (lines 19-21 and 115), referring the reader to theoretical literature works for further insight.

6. Gaussian detrending: what bandwidth (standard deviation of the Gaussian filter) was taken? Depending on the bandwidth of the Gaussian filter, it is not surprising that the Gaussian filter is highly correlated to the linear moving average filter (on line 315).

We agree with Reviewer #1 that the correlation does not come with great surprise, as the bandwidth was taken to be 7 days in order to reflect known weekly fluctuations related to testing routines. The discussion has been included in line 369.
Also line 194, Dessavre et al., don’t recommend Gaussian detrending (citation [46]) In fact, they found that EWSs can exhibit odd behaviours when a moving window method is used to detrend the data.
We agree that Dessavre et al. observe altered behaviors when using moving window methods and suggest using replicates. The replicates method is, unfortunately, unfeasible with such real-world time series as the candidate samples show different features. We thus followed Dessavre et al’s suggestion to test Gaussian windows (from Dakos, 2008 [now properly referenced]) and to compare different detrending methods and moving window sizes to recognize consistent trends. To complete the manuscript, this important piece of discussion has thus been now included among the limitations of the study (line 563).

7. Can table 2 be written as |AUC - 0.5|. As you pointed out, an AUC score of 0 means that it can be perfectly classified by its negative trend, so CV and variance seem to be classified very well for the N data set - could you discuss what this might tell us.
   We thank Reviewer #1 for the suggestion, but we believe that keeping the absolute value of the AUC is more relevant. In fact, it stresses that a good detection on the N data set can be obtained with decreasing trends of variance and CV – which is unexpected and contradicts the theoretical predictions. In fact, after Reviewer #1’s remark, we have further investigated why this could happen. One of the following situations could apply: a continuation of a decreasing trend after the first wave or fluctuations is a rather stable trend, eventually followed by a delayed increase after the transition. We hypothesize that these features are again associated with the rapid re-emergence and the noise distribution. On the one hand, the indicator had little time to settle to stable trends and then increase again; on the other hand, there could be some bifurcation delay playing a role. Hence, we suggest that this is a spurious effect that should be treated carefully. We have now included this discussion in the text (lines 489-494) and we have expanded the supplementary material (Sec S7 and Fig S6) to incorporate this additional analysis.

Please notice that we have also corrected Table 2: a rounding mismatch was giving AUC = 0 for Var and CV, while the true values are slightly higher and do not impair the discussion above.

8. AC(1) is very noisy and using a short time series is not recommended

We agree with Reviewer #1. We have included this remark in the text (line 384).

9. In figure 1b, can the excess kurtosis be calculated instead (Kurt - 3). This would make it easier to read this figure.
   We thank Reviewer #1 for the good idea, which has now been implemented.

**Reviewer #2**

The authors use recent COVID-19 data from multiple countries to perform a careful analysis of the performance of EWS at detecting resurgent outbreaks. As the authors state, there has been limited studies attempting to quantify EWS performance using actual epidemiological data (rather than simulated data).

I enjoyed reading the paper, and found the overall study was carefully designed and well-motivated. I found the paper well-suited to PLoS Computational Biology, however there were some points in the methods I remain unclear on and I do have some concerns with some decisions made in the analysis.

Provided these issues are addressed I would be willing to recommend accepting the paper.

We thank Reviewer #2 for the positive feedback and we hope we have now addressed all the concerns expressed.

Major issues:

Data:

The authors calculate the EWS from the number of “active cases” in the population. Active cases are calculated from epidemiological data as the difference between cumulative confirmed cases and cumulative deaths plus confirmed recovered (Eq. 4). However, my experience of COVID-19 data is that the “recovered cases” dataset is usually highly incomplete: Many individuals test positive then recover at home with no further medical system contact. Using Eq. 4, these missing recoveries correspond to an infinite infectious period — i.e. the error in estimated active cases will accumulate over time and may substantially differ from the number of active cases. At an extreme, this accumulating error might give the illusion of re-emergence in locations where it might not have occurred. Unless the authors are confident that robust public health protocols are in place for following up on the infectious status of all confirmed cases for every country studied, I strongly advise not calculating active cases in this way.

I appreciate the issue the authors are trying to resolve: That most of the theory is for prevalence data (as the authors explain in the supplementary material). However I’m worried the active cases approach introduces more problems than it solves. If missing recoveries are an issue I suggest calculating the EWS using incidence data (such as daily confirmed cases), see Refs. 15 and 16 for details.

We acknowledge the concerns raised by Reviewer #2, which we have addressed in two ways: constructing a curated dataset for prevalence data, in order to minimise the chance of severe biases, and re-running the analysis on incidence data to provide a complete comparison.

First, we acknowledge that estimates of recovered cases may be biased due to limited tracking of such patients. However, we have tried our best to limit the biases associated with the used data. As described in Sec. 2.2, we curated the dataset following several quality criteria. We did not consider all countries displaying clear deviations from expected epidemic trends in their proxy *A* (e.g. “saw-like” curves or not decreasing curves like the ones displayed in Fig. S2); during the data analysis, these were in most parts associated with biased reported recovered cases, for instance reported all in one day. We did not consider those countries with a share of positive tests > 5% close to the transition, to limit as much as possible the effects of undertesting (the cut-off has been chosen in accordance with WHO guidelines and is how we account for the number of tests taken). We also did not consider regional areas where the assumptions of homogenous mean-field diffusion clearly do not hold (e.g. where spacial heterogeneity is recognized in the literature, or when there were very few cases over the whole considered time period, mostly representative of stochastic fluctuations).

Luxembourg is a different case: since the country is small and the hospital system was carefully monitored, we are more confident about the numbers reported. Consequently, we have first concentrated on this country for the initial comparisons of empirical analysis and theoretical predictions.

In order to compare our results to several literature studies (including those specifically dedicated to critical transitions in general), we have first focused on this aspect throughout the text. Nonetheless, we agree with Reviewer #2 that more recent studies have considered the expected behavior of incidence data, and that, despite our efforts to minimize the impact of biases in our proxy for *A,* we cannot rule them out completely. Hence, we followed Reviewer #2’s suggestion to repeat the analysis on incidence data, which supports the findings obtained from the previous analysis. It is now included and discussed in the text (lines 192-200 and 579-597) and all the corresponding results have been included in electronic supplementary information (Section S8).

Bayesian inference:

I’m confused how the Bayesian inference was performed (after reading both main manuscript supplementary information), and think the section in the main manuscript needs to be a lot more comprehensive. E.g. was a rolling window (from *t* to *T*) used? Was the window centred? What MCMC algorithm was used?
We apologize for our description not being clear. We have now incorporated a more comprehensive description in the main text (lines 205-213) and more details in the supplementary material. The estimation is performed day by day when new data are available, and corresponds to calculating the likelihood of observing k cases, given a candidate R(t) and following a Poisson transmission model. To avoid fitting spurious bumps, the data were previously smoothed with a centered Gaussian kernel over 7 days. Note that, since this is only used for a retrospective analysis, it does not modify the non-anticipating scheme for the EWS. To generate candidate R(t), we used a Metropolis-Hastings algorithm. Our implementation expands Systrom et al. (2020), to which we also refer for further details.

Also how exactly was $t_{em}$ determined? Its unclear to me whether it was the time of the first maximum of $P(R(t)>1)$ or whether when $P(R(t)>1)=1$.

We apologize with Reviewer #2 for not being sufficiently clear in the text. In our definition, $t_{em}$ corresponds to the first time when P(R(t)>1) reaches its maximum value of 1. The text was edited accordingly (lines 214-218).

Timeseries detrending:

While the estimator of the variance (Eq. 7) is careful to ensure that the variance at time *t*, ${Var}_{i,t}$, only depends on data up to time *t*, i.e. it is non-anticipating, I’m unclear if this is also the case for all the detrending methods (e.g. ARIMA and Gaussian kernel). If removing the trend at time *t* relies on data “from the future” then it becomes hard to assess EWS performance at detecting emergence.

We confirm that all methods are non-anticipating. This aspect has now been clarified in the text (lines 257-259). In particular, our algorithm specifies that the Gaussian kernel is not centered, but associated to the rightmost point of the window. The ARIMA is run on data only up to the desired point: this gives slightly poorer results at the beginning when there are few datapoints from the past, but soon improves. We remark that this is not an issue as the initial datapoints still refer to the first wave and are not considered for EWS calculation.

ROC analysis:

Lines 251-256 left me unclear how exactly the ROC curve was calculated, i.e. exactly which data (both time range and which countries) were used to calculate Kendall’s tau for the test and null cases. One issue identified in Ref. 18 is that an initial downward trend in incidence (before resurgence) can mask the ability of EWS to detect resurgence. Was this an issue for any of the countries studied (it’s hard to know without details on what dates were used for EWS calculation and Kendall’s tau calculations)?

We thank Reviewer #1 for this opportunity to make our text clearer. First, we compute each indicator over rolling window of 14 days, starting from 30 days before the transition (long enough to have several data points, but not too long to overlap with the first wave) up to 5 days after the transition (to account for possible small bifurcation delays). These were the test cases. For the null cases, we use τ values taken long before the transition occurs, that is on windows associated with timepoints t<-30. We compare each statistical indicator’s ability to correctly distinguish which Kendall's tau scores belong to those from before or after re-emergence, that is, we determine whether the estimated tau is higher or lower than some threshold value at that timepoint and determine whether each simulation is classified correctly by that threshold. This gives a proportion of true positives and false positives. To do so, we compare various values from 0<τ<1 to those of the positive and negative data set, for each country. We calculate the indicator for each country in a test set at the given timepoint, and then group the specificity and sensitivity results to obtain the final ROC curve.

We have now edited the text to incorporate this extended explanation in lines 284-300 (sec 2.5).

A downward trend before resurgence was observed in countries belonging to the N dataset, and indeed is likely masking the ability of EWS to detect the transition. This aspect is now discussed in lines 489-494 and in Supplementary Material Sec S7.

Figures:

Figure 1: State which country the example time series (panel a) corresponds to.

We thank Reviewer #2 for noticing the incorrect removal of the country’s reference. It has now been re-included in the caption of Fig. 1.

Figure 2: Formatting error of first sentence. State which time series is being detrended and shown in panels a b and c (is in Luxembourg and the same as Figure 1a?).

We confirm that the figure refers to Luxembourg. The figure caption has now been edited appropriately.

I’m surprised the ARIMA and Gaussian are so poorly correlated. Do you have any explanation why?

The ARIMA method introduces more complex time-series analysis (in addition to Moving Average, it employs an Integration term and an AutoRegressive method). Hence, we hypothesize that it might better capture short-term patterns (like a weekend effect associated with lower testing rates) and provide richer descriptions. Lines 408-417 (now lines 474-483) of the previous manuscript version also extend this point.

Do you know why the residuals around the centre are so much smaller than the others?

The residuals around the mean are usually larger in magnitude when the mean is also larger. As the center of the figure relates to a period with few cases (almost zero, used as initial condition before the re-emergence), the residuals are similarly smaller in magnitude.

Also it’s hard to relate the x axis (date points) to the x axis of other figures — maybe stick to date like Figure 1 and 3

We thank Reviewer #2 for his/her suggestion, which has now been implemented.

Figure 5: The caption confused me (for the same reasons as the section on the ROC and needs reworking). I also don’t understand how the curves could represent averages over counties in the test set? Are the value of tau for each country not the samples used in calculating the ROC curve?

We thank Reviewer #2 for pointing out this issue. Indeed, we did not calculate an “average”. Rather, we calculate the indicator for each country at the given timepoint, and then group the specificity and sensitivity results to obtain the final ROC curve. This explanation is now included in the text line 297 and in the referenced caption.

Minor issues:

Line 27: Sentence unclear: “If this would be the case, the consequences would be far-reaching, possibly expanding the set of epidemic indicators, using complexity theory.”

Line 141: Type: “based on the theory recalled above theory and”

Lines 151-175: I’m not sure on the journals style but if possible it would be better to format data sources as reference rather than URLs in the main text.

Line 167: Typo in “country-wide”

We thank Reviewer #2 for the careful proofreading. We have corrected the text where suggested, along with a few other typo corrections. Regarding the question about URLs, we did not find strict guidelines on the journal webpage, but we will make sure with the editorial team which style is preferred.
